# Effects of Transcranial Direct Current Stimulation over the Primary Motor Cortex in Improving Postural Stability in Healthy Young Adults

**DOI:** 10.3390/biology11091370

**Published:** 2022-09-17

**Authors:** Jinqian Hou, Michael A. Nitsche, Longyan Yi, Zhaowei Kong, Fengxue Qi

**Affiliations:** 1 Key Laboratory of Sport Training of General Administration of Sport of China, Beijing Sport University, Beijing 100084, China; 2 Sports, Exercise and Brain Sciences Laboratory, Beijing Sport University, Beijing 100084, China; 3 Department of Psychology and Neurosciences, Leibniz Research Centre for Working Environment and Human Factors, 44139 Dortmund, Germany; 4 China Institute of Sport and Health Science, Beijing Sport University, Beijing 100084, China; 5 Faculty of Education, University of Macau, Taipa, Macau 999078, China

**Keywords:** non-invasive brain stimulation, single-leg stand, jump-landing task, static postural stability, dynamic postural stability, postural balance control

## Abstract

**Simple Summary:**

Transcranial direct current stimulation (tDCS) is used as an adjuvant rehabilitation treatment to improve postural control and lower limb function. This study explored the effects of sham and anodal tDCS over the leg region of M1 on static and dynamic postural stability in healthy young adults. Jump-landing tasks were used to examine dynamic postural stability. Static postural stability was assessed by a single-leg stand on force plates with open eyes. Anodal tDCS had an immediate improving effect on static and dynamic postural stability, and might evolve as an adjuvant rehabilitation treatment to enhance postural deficits in the future.

**Abstract:**

Transcranial direct current stimulation (tDCS) over the primary motor cortex (M1) is of increasing interest to improve motor performance in healthy adults and patients with respective deficits. This study aimed to examine whether tDCS over M1 can improve static and dynamic postural stability in young healthy adults. Seventeen healthy participants (mean age = 25.14 ± 2.50 (standard deviation, SD) years) received sham and anodal tDCS (2 mA) over the vertex at the Cz electrode position for 15 min. Static and dynamic postural stability were evaluated before and immediately after tDCS. The center of pressure (COP) sway area (COP_SA_) and COP maximum displacements to medio-lateral (COP_ML_) and antero-posterior directions (COP_AP_) were used to evaluate static postural stability. The anterior–posterior stability index (APSI), medial–lateral stability index (MLSI), vertical stability index (VSI), dynamic postural stability index (DPSI), and time to stabilization (TTS) in forward (FL), 45° anterior lateral (LL), and 45° anterior medial (ML) direction landing, as well as the Y-balance composite score (YBT_CS_) were used to assess dynamic postural stability. The results showed that the LL-TTS (*p* = 0.044), non-dominant leg COP_SA_ (*p* = 0.015), and YBT_CS_ (*p* < 0.0001) were significantly improved in the real stimulation as compared with the sham stimulation session, and anodal tDCS significantly changed dominant leg COP_AP_ (*p* = 0.021), FL-APSI (*p* < 0.0001), FL-TTS (*p* = 0.008), ML-TTS (*p* = 0.002), non-dominant leg YBT_CS_ (*p* < 0.0001), and dominant leg YBT_CS_ (*p* = 0.014). There were no significant differences in all obtained balance values in the sham stimulation session, except for non-dominant leg YBT_CS_ (*p* = 0.049). We conclude that anodal tDCS over M1 has an immediate improving effect on static postural stability and dynamic performance in young healthy adults. This makes tDCS a promising adjuvant rehabilitation treatment to enhance postural stability deficits in the future.

## 1. Introduction

Postural stability is essential for almost all aspects of daily life, especially in maintaining normal physiological activities, such as walking, running, and jumping. Dynamic postural stability, which is defined as maintaining balance ability while transitioning from a dynamic process to a static position, is fundamental for optimal motor performance [1]. It includes the ability to control ground reaction force and orientation [2]. Static postural stability is the ability to maintain a steady standing posture on a static base of support [3]. Neurophysiological and imaging studies have demonstrated that the primary motor cortex (M1), supplementary motor area, premotor cortex, prefrontal cortex, visual cortex, basal ganglia, cerebellum, and brainstem are involved in human balance control [4,5,6,7]. The central nervous system integrates multi-sensory information from visual, vestibular, and proprioceptive sensory sources, amongst others, to maintain postural stability in a dynamic environment [8,9]. Postural stability deficit occurs at high rates of recurrent injury in daily life activities. Optimizing the interventions to enhance postural stability is of the utmost importance for rehabilitation programs.

Transcranial direct current stimulation (tDCS) is a non-invasive brain stimulation tool suited to modulating cortico-spinal excitability and altering motor functions through a constant weak direct current which enters the brain via the skull [10]. These currents alter neuronal membrane polarization. At the macroscale level, stimulation with the anode placed over the motor cortex with conventional electrodes enhances cortical excitability [11,12] during, but also after stimulation, if stimulation is sufficiently dosed. The after-effects of tDCS resemble features of neuroplasticity [13]. It was reported that a single session of atDCS over M1 has a positive effect on postural stability in young adults. Dutta et al. showed that atDCS over M1 for 10 min improved static postural stability during quiet stance with eyes closed and that this effect was accompanied by an increase in corticospinal excitability in healthy young adults [14]. Moreover, atDCS over M1 for 15 min decreased the center of pressure (COP) in the medio-lateral direction and the COP sway area as compared with sham tDCS in healthy young adults [15]. Kaminski et al. showed that atDCS over M1 for 20 min improved dynamic balance task performance in healthy young adults [16]. Baharlouei et al. showed that atDCS over M1 for 20 min improved static balance in healthy older adults [17]. The authors speculated that this might be caused by an atDCS-induced enhancement of spinal network excitability and cortico-muscular coherence in ankle muscles, as well as increased lower limb muscle tone, muscle stiffness [14,18], and locomotor adaptation [13]. In addition, several studies reported that atDCS combined with physical training increased M1 excitability and dynamic balance performance in patients with chronic ankle instability (CAI) [19,20], and one of the major mechanisms for these effects might be activation of the cortical representation of the peroneus longus muscle, which promoted recruitment of fast motor units [19,21]. Taken together, these results indicate that M1 is closely related to postural control [22]. However, some studies reported different results, suggesting that atDCS over M1 does not improve static and dynamic balance in healthy young adults as compared with sham stimulation [23,24]. Kaminski et al. also found tDCS over the M1 leg area improved dynamic balance learning in young adults but not in healthy older adults as compared with sham stimulation [25]. A possible reason for this might be that in these studies tDCS did not effectively alter the connection between the motor cortex and muscles. These differences of results between studies might be caused by different study protocols, including outcome measures, and different levels of physical activity of the study populations included. 

To our knowledge, previous studies have showed that postural stability was assessed by a balance system in standing on one foot or both feet [26,27,28]. In this study, we used the complex lower limb motor tasks to systematically evaluate postural stability to further verify whether tDCS over M1 could improve the postural stability. Jump-landing tasks were used to simulate physical activity to examine dynamic postural stability. Center of pressure (COP) parameters were used to assess static postural stability by single-leg standing with eyes open. We examined the effects of sham and atDCS over the leg region of M1 (Cz) on static and dynamic postural stability in healthy young adults. We hypothesized that atDCS would enhance static and dynamic postural stability in comparison with sham stimulation.

## 2. Materials and Methods

### 2.1. Participants 

A total sample size of 13 healthy participants resulted for an effect size (f = 0.35) [16,17] with 80% power and a 5% level of statistical significance via G*Power 3.1.9.2 software [29,30]. Twenty-one participants were recruited to participate in this study, and 4 participants dropped out after completing the first session. Seventeen healthy adults (10 females and 7 males, mean age 25.14 ± 2.50 (standard deviation (SD) years, 143.12 ± 78.88 (SD) mins of exercise regularly per week) completed this study and received sham and anodal tDCS with an interval of at least 3 days. Exclusion criteria included any history of neurological or psychiatric diseases, and lower extremity injury 6 months before tests. Informed consent was obtained from each participant prior to participation in the study. This study was approved by the ethics committee of Beijing Sport University and the study meets the standards of the Declaration of Helsinki (registration No. 2021049 H).

### 2.2. Experimental Procedure

**Figure 1:**
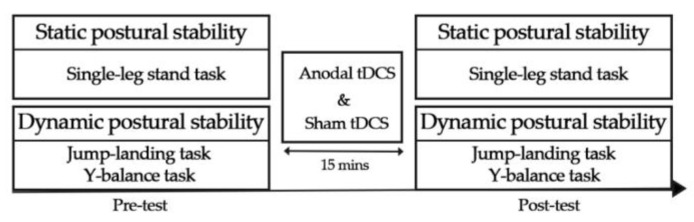
Flow of the experimental procedure.

This study was conducted using a single-blind, within-subjects design. Each participant visited the laboratory twice, with an interval of at least 3 days. Each visit consisted of static and dynamic postural stability tests (single-leg stand, jump-landing tasks and Y-balance test) before and immediately after 15 min of either anodal tDCS or sham stimulation in a randomized order (Figure 1). In order to avoid fatigue, participants were asked not to conduct strenuous exercises during the whole experimental procedure. Participants were not to consume alcohol or coffee the day before the experimental sessions. In order to avoid distraction, this study was conducted in a relatively quiet place. Before the test, participants completed a 15 min warm-up (jogging, jumping, and stretching). The single-leg stand test, jump-landing tasks, and Y-balance test (YBT) trials were conducted in counter-balanced order before and immediately after atDCS or sham stimulation. The duration of the test tasks was approximately 31 min after atDCS or sham stimulation. 

### 2.3. Static Postural Stability Test 

The single-leg stand test was performed to assess static postural stability. COP parameters were measured by force plates (1000 Hz, Kistler, 9281 CA, Switzerland), a method for static postural stability assessment [31]. Participants were instructed to stand upright on the force plate on the dominant or non-dominant leg alternately and gaze at a visual target 3 m in front of them. During the test, participants kept their hands on their pelvis and held the non-stance leg flexed at about 45°. In each trial, participants stood on one leg for 30 s, followed by rest for 30 s, and both the dominant and non-dominant leg were alternated 3 times. A test was considered a failure if the non-stance leg touched the ground or an arm was moved away from the pelvis. 

### 2.4. Dynamic Postural Stability Test 

Dynamic postural stability was assessed by three jump-landing tasks and the Y-Balance test. Three jump-landing directions were defined as forward landing (FL), 45° anterior lateral landing (LL), and 45° anterior medial landing (ML) [32]. Participants put their hands on their pelvis and stood on their dominant leg, located at a 30 cm distance from the center of the force plate. They were instructed to jump over a 10 cm obstacle and land in the middle of the force plate, and then to stabilize the whole body as quickly as possible and keep their balance for at least 10 s. Participants performed each direction of landing in the order of FL, LL, and ML, and rested for 30 s between trials. The criteria for a trial failure and repetition of the trial were (1) the passive limb touched the floor; (2) the landing foot did not point forwards, and movements after landing; or (3) hands were moved away from the pelvis to support body balance. Three successful trials of the dominant leg were performed for each direction. The dominant leg was determined by the participants by choosing with which leg they preferred to use to kick a ball [33]. 

The Y-balance test (YBT) is extensively used to assess dynamic postural stability [34]. Participants were allowed to practice 3 times prior to formal testing in the anterior (A), postero-medial (PM), and postero-lateral (PL) directions. Participants stood barefoot on a single leg and put their big toe on the center of a grid. They then put their hands on their pelvis and moved the other foot to reach the maximal distance in each direction, and then returned it to the starting position [35]. The maximum reach distance to the nearest 0.5 cm was recorded. The criteria of a trial failure and repetition of the trial were (1) the reaching foot touched the ground, (2) the participant took their bodyweight on the reaching foot, (3) the reaching foot failed to return to the starting position, or (4) the hands failed to stay on the pelvis. Three successful trials were collected in each direction. The YBT composite score (YBT_CS_) was calculated by averaging the individual leg-length normalized scores for each direction [36]. The leg length was recorded by measuring the distance between the anterior–superior iliac spine and the medial malleolus [37].

YBT_CS_ = [(A + PM + PL)/(leg length × 3)] × 100

### 2.5. tDCS 

tDCS was delivered by a constant direct current stimulator (Yingchi, TCS-E2, Shenzhen, China) using a pair of humidified sponge electrodes (0.9% NaCl, 35 cm^2^). The anodal electrode was placed on the scalp over the leg area of M1 (Cz). The distance between the pre-auricular points crossing the vertex of the head and the distance from the nasion to the inion were measured, and the middle position was marked as the Cz region [38], in accordance with the 10–20 EEG international system. The cathodal electrode was placed on the forehead medial above the nasion. atDCS was applied for a duration of 15 min with 30 s of ramp-up and ramp-down, with a current intensity of 2 mA. For sham stimulation, the current was delivered for 30 s and then turned off [39,40]. 

### 2.6. Data Analysis

In static postural stability, COP parameters included COP sway area (COP_SA,_ mm^2^, 95% confidence ellipse), COP maximum displacements in medio-lateral directions (COP_ML_) (mm), and COP maximum displacements in antero-posterior directions (COP_AP_) (mm).

Dynamic postural stability were measured by the anterior-posterior stability index (APSI) (1), medial-lateral stability index (MLSI) (2), vertical stability index (VSI) (3), and dynamic postural stability index (DPSI) (4). They were calculated using the first 3 s of the ground reaction forces (GRF) after initial ground contact (GRF ≥ 10 N) [41,42]. W represents the participant’s body weight. GRFx and GRFy refer to the horizontal components of the force plate and GRFz refers to its vertical component [43]. This method has good test-retest reliability with intraclass correlation coefficients (ICC) of 0.86–0.90 and standard errors of mean (SEM) between 0.028 and 0.06 [44,45]. Higher stability indices and DPSI values represent worse dynamic postural stability [46]. In addition, time to stabilization (TTS) was quantified as the first time-point at which vertical GRF reached and remained within ± 5% of the body weight for at least 1.0 s after initial ground contact [47]. TTS refers to the shortest time of stabilizing the body sway in three jump-landing directions [45,48]. A longer TTS indicates poorer dynamic postural stability [33,42].

(1) $$APSI=\frac{\sqrt{\sum {\left(0-GRF_{x_{i}}\right)}^{2}/n}}{w}$$

(2) $$MLSI=\frac{\sqrt{\sum {\left(0-GRF_{y_{i}}\right)}^{2}/n}}{w}$$

(3) $$VSI=\frac{\sqrt{\sum {\left(w-GRF_{z_{i}}\right)}^{2}/n}}{w}$$

(4) $$DPSI=\frac{\sqrt{\left[\sum {\left(0-GRF_{x_{i}}\right)}^{2}+\sum {\left(0-GRF_{y_{i}}\right)}^{2}+\sum {\left(w-GRF_{z_{i}}\right)}^{2}\right]/n}}{w}$$

Data were analyzed using a WOLFRAM MATHEMATIC software (Wolfram Research, Inc., Mathematica Trial Chinese Edition, Version 13.1, Champaign, IL, USA). The COP parameters and GRF data were low-pass filtered with a cut-off frequency of 10 Hz by a digital 4th-order Butterworth filter [43]. All data were averaged across the three successful trials completed by each participant.

### 2.7. Statistical Analysis 

All data were analyzed by SPSS (SPSS IBM., Chicago, IL, USA, version.26.0) and were presented as mean ± SD. The normal distribution of the data was evaluated by Shapiro–Wilk tests. Two-way repeated-measures analyses of variance (ANOVAs) with the within subject factors session (sham tDCS and anodal tDCS), and time (before/after intervention) were used for the dependent variables COP_SA_, COP_ML_, COP_AP_, APSI, VIS, MLSI, DPSI, and YBT_CS_. Fisher’s post hoc tests were used to determine differences between sessions as well as before and after intervention in case of significant ANOVA results. The effect size was calculated for all ANOVAs using partial eta-squared (η*_p_*^2^). The significance threshold was set at α = 0.05.

## 3. Results

All participants completed this study and tolerated tDCS well. The repeated-measures ANOVA showed no significant differences in baseline measurement in COP_SA_, COP_AP_, COP_ML_, APSI, MLSI, VSI, DPSI, TTS, and YBTcs between the two experimental sessions (all values of *p* > 0.05). The results of repeated-measures ANOVAs are shown in Table 1. The means (SD) of static and dynamic postural stability tests are shown in Table 2. 

### 3.1. Static Postural Stability 

Regarding COP_SA_ of the non-dominant leg, the repeated-measures ANOVA showed a significant interaction effect between time and session (*F*_(1, 16)_ = 5.146, *p* = 0.037, η*_p_*^2^ = 0.243), but the main effects of time and session were not significant. Fisher’s LSD post hoc tests revealed a significant decrease in the atDCS session after intervention as compared with the sham stimulation session (*p* = 0.015). Within the atDCS session, COP_SA_ of the non-dominant leg significantly decreased post intervention in contrast with baseline (*p* = 0.018), but did not significantly differ in the sham stimulation session before and after an intervention. For COP_SA_ of the dominant leg, the analysis showed a significant interaction effect between time and session (*F*_(1, 16)_ = 7.025, *p* = 0.017, η*_p_*^2^ = 0.305), but the main effects of time and session were not significant. The post hoc tests, however, showed no significant differences between the atDCS and sham stimulation sessions, and no significant differences were shown between pre- and post-test conditions within the atDCS and sham stimulation sessions. 

Regarding COP_AP_ of the dominant leg, a significant main effect of time emerged (*F*_(1, 16)_ = 4.759, *p* = 0.044, η*_p_*^2^ = 0.229), but the main effect of session and the interaction effect between time and session were not significant. Fisher’s LSD post hoc tests revealed a significant decrease between pre-test and post-test in the atDCS session (*p* = 0.021), but not in the sham stimulation session. For COP_AP_ of the non-dominant leg and the bilateral COP_ML_, the repeated-measures ANOVAs revealed no significant main effects for the factors of time, session, or the interaction between time and session. 

### 3.2. Dynamic Postural Stability

For the forward landing task, in the FL-APSI, the repeated-measures ANOVA showed a significant main effect of time (*F*_(1, 16)_ = 27.423, *p* < 0.001, η*_p_*^2^ = 0.632), and interaction between time and session (*F*_(1, 16)_ = 5.013, *p* = 0.040, η*_p_*^2^ = 0.239), but the main effect of session was not significant. Fisher’s LSD post hoc tests showed a significant decrease between pre-test and post-test in the atDCS session (*p* = 0.000142), but not in the sham stimulation session. Regarding FL-TTS, a significant main effect of time emerged (*F*_(1, 16)_ = 6.145, *p* = 0.025, η*_p_*^2^ = 0.277), but the main effect of session and the interaction between time and session were not significant. The post hoc tests revealed a significant decrease from pre-test to post-test in the atDCS session (*p* = 0.008), but not in the sham stimulation session. For FL-MLSI, FL-VSI, and FL-DPSI, the repeated-measures ANOVAs showed no significant main effects of time, session, or interaction between time and session.

In the 45^°^ anterior lateral landing task, for LL-APSI, a repeated-measures ANOVA showed a significant main effect of time (*F*_(1, 16)_ = 6.089, *p* = 0.025, η*_p_*^2^ = 0.276), but the main effect of session and the interaction between time and session were not significant. The post hoc tests showed a significant decrease from pre-test to post-test in the atDCS session (*p* = 0.016) but not in the sham stimulation session. For LL-TTS, the main effect of time (*F*_(1, 16)_ = 7.976, *p* = 0.012, η*_p_*^2^ = 0.333), and the interaction between time and session (*F*_(1, 16)_ = 9.698, *p* = 0.007, η*_p_*^2^ = 0.377) were significant, but the main effect of session was not significant. Fisher’s LSD post hoc tests revealed a significant decrease between atDCS session and sham stimulation session (*p* = 0.044) and significantly decreased post-test as compared with pre-test values in the atDCS session (*p* = 0.004), but not in the sham stimulation session. For LL-MLSI, LL-VSI, and LL-DPSI, the repeated-measures ANOVAs showed no significant main effects of time, session, or interaction between time and session.

Regarding ML-TTS, the results showed a significant interaction effect between time and session (*F*_(1, 16)_ = 6.524, *p* = 0.021, η*_p_*^2^ = 0.290), and a significant main effect of time (*F*_(1, 16)_ = 8.086, *p* = 0.012, η*_p_*^2^ = 0.336), but not session. The post hoc tests revealed a significant decrease between pre-test and post-test in the atDCS session (*p* = 0.002), but not in the sham stimulation session. In the 45^°^ anterior medial landing task, for ML-APSI, ML-MLSI, ML-VSI, and ML-DPSI, the results showed no significant main effects of time, session, or interaction between time and session.

In the Y-balance tests, for YBT_CS_ of the dominant leg, the repeated-measures ANOVA revealed a significant main effect of time (*F*_(1, 16)_ = 10.159, *p* = 0.006, η*_p_*^2^ = 0.388), but the main effect of session and the interaction between time and session were not significant. Fisher’s LSD post hoc tests revealed significant improvement between pre-test and post-test in the atDCS session (*p* = 0.014), but not in the sham stimulation session. Regarding YBT_CS_ of the non-dominant leg, the analysis showed a significant interaction between time and session (*F*_(1, 16)_ = 71.372, *p* < 0.0001, η*_p_*^2^ = 0.812) and significant main effects of time (*F*_(1, 16)_ = 148.075, *p* < 0.0001, η*_p_*^2^ = 0.902) as well as session (*F*_(1, 16)_ = 18.198, *p* = 0.001, η*_p_*^2^ = 0.532). Fisher’s LSD post hoc tests showed significant improvement after intervention in the atDCS session in contrast with the sham stimulation session (*p* < 0.001) and between pre- and post-test in the atDCS (*p* < 0.001) as well as sham stimulation sessions (*p* = 0.049).

## 4. Discussion

This study investigated whether a single session of tDCS over the leg region of M1 could affect static and dynamic postural stability in healthy young adults in comparison with sham stimulation. In accordance with our hypotheses, anodal tDCS improved static and dynamic postural stability in healthy young adults. 

In detail, anodal tDCS over the leg region of M1 decreased COP_SA_ of the non-dominant leg in comparison with sham stimulation and reduced non-dominant-leg COP_SA_ and dominant-leg COP_AP_ from pre-test to post-test in the atDCS session. COP_SA_, COP_AP_, and COP_ML_ are commonly considered as important parameters of the stability of human standing posture. Our study is in line with previous findings that atDCS decreases the COP sway area and improves postural control ability [14,49]. Dutta et al. showed that atDCS decreased the COP sway area and COP position in the medio-lateral direction during quiet stance and increased corticospinal excitability in healthy adults [14,15]. Furthermore, Baharlouei et al. showed that atDCS over M1 improved static balance in healthy elderly persons [17]. Maintaining postural stability requires integration of different sensory systems, including visual, vestibular, and somatosensory information to perform motor adjustments [50]. It involves several brain areas, specifically the frontal cortico-basal ganglia network, including the primary motor cortex, premotor and supplementary motor cortex, and basal ganglia (putamen) [5,51]. On the other hand, functional near-infrared spectroscopy (fNIRS) showed that sensorimotor cortical areas were activated in static balance control [50], and atDCS over the left sensorimotor cortex modulated the cortical responsiveness to control foot pressure stimuli in healthy young adults [52]. This might indicate that atDCS over Cz promoted cortical spinal excitability and improved the sensorimotor integration to modulate motor output, and then increased cortico-muscular coherence of the tibialis anterior muscle [14] and lower limb muscle tone and stiffness to maintain the COP within the base of support in maintaining postural stability [14,18,53]. 

In the dynamic postural stability test, the results showed that tDCS improved the LL-TTS and non-dominant YBT_CS_ in the atDCS session as compared with sham stimulation, decreased the FL-APSI and FL-TTS as well as ML-TTS, and increased non-dominant leg and dominant leg YBT_CS_ between pre-test and post-test in the atDCS session. These results are in accordance with several studies showing that atDCS over M1 improves the dynamic balance learning in healthy adults [16] and dynamic balance in individuals with postural disorders [19]. The current study expanded this knowledge to healthy young adults. Functional magnetic resonance imaging (fMRI) study showed that locomotor-related networks (e.g., M1, supplementary motor area, pre-motor cortex) are activated in complex balance tasks [5]. Several studies showed that tDCS over M1 improves motor cortex excitability and alters motor unit firing rates [54], increases lower limb strength [55,56,57], and enhances locomotor adaption aftereffects [13]. The dynamic postural stability indices and TTS in the current study mainly reflect the degree of the center of mass (COM) deceleration and COM oscillation control [2], and it is relevant to muscle stiffness [2,58]. The improvement of dynamic postural stability might be caused by a quick deceleration of the oscillation of the COM within the area of the base of support, which might be determined by muscle strength and ankle proprioception [59]. Therefore, tDCS might modulate these relevant factors to maintain postural stability in a dynamic process. 

In addition, it is worth mentioning that there were higher scores of APSI and DPSI in people with a history of a lateral ankle sprain and CAI compared with the healthy group [60]. Our results were in line with one study that atDCS combined with eccentric exercise can improve dynamic postural stability in comparison with the sham stimulation group in patients with CAI [19]. This implies that tDCS improves dynamic postural stability and has a potential benefit in injury prevention in healthy young adults, and tDCS can be used as an adjuvant rehabilitation approach in patients with CAI.

## 5. Limitations of the Study

Some limitations of this study should be taken into consideration. This study used a single-blind, within-subjects design and lacked a blank control session. Further research should apply multi-session instead of a single session tDCS interventions to explore long-term effects of stimulation on static and dynamic postural stability, since in the present study we explored only acute effects. As the differences between dominant and non-dominant legs were not considered, only the dominant leg was examined in the jump-landing task of the dynamic postural stability indices. Further research could verify the effect of tDCS in improving the dynamic postural stability of the bilateral limbs. The current study included only behavioral parameters, and lacked electroencephalographic recordings, which would have been helpful to understand mechanisms of tDCS-improved static and dynamic postural stability. 

## 6. Conclusions

Single-session anodal tDCS was effective in improving static postural stability and dynamic performance in healthy young adults. This preliminary evidence might help to develop tDCS as an adjuvant preventive, and a therapeutic approach to enhance functional rehabilitation. 

## Figures and Tables

**Table 1:** Results of the repeated-measures ANOVAs for static and dynamic postural stability.

Test	Parameters	Factor	*df*	*F* Value	*p* Value	η*_p_*^2^
Static Postural stability	non-COP_SA_	session	1, 16	2.901	0.108	0.153
		time	1, 16	0.261	0.616	0.016
		time × session	1, 16	5.146	0.037	0.243
	dom-COP_SA_	session	1, 16	0.186	0.672	0.012
		time	1, 16	0.251	0.623	0.015
		time × session	1, 16	7.025	0.017	0.305
	non-COP_AP_	session	1, 16	0.474	0.501	0.029
		time	1, 16	1.752	0.204	0.099
		time × session	1, 16	0.016	0.900	0.001
	dom-COP_AP_	session	1, 16	2.889	0.109	0.153
		time	1, 16	4.759	0.044	0.229
		time × session	1, 16	3.505	0.080	0.180
	non-COP_ML_	session	1, 16	0.017	0.899	0.001
		time	1, 16	0.048	0.829	0.003
		time × session	1, 16	0.244	0.628	0.015
	dom-COP_ML_	session	1, 16	0.129	0.724	0.008
		time	1, 16	1.202	0.289	0.070
		time × session	1, 16	1.075	0.315	0.063
Forward Landing task	FL-APSI	session	1, 16	0.303	0.590	0.019
		time	1, 16	27.423	0.0001	0.632
		time × session	1, 16	5.013	0.040	0.239
	FL-MLSI	session	1, 16	2.238	0.154	0.123
		time	1, 16	0.073	0.790	0.005
		time × session	1, 16	0.003	0.956	0.0001
	FL-VSI	session	1, 16	1.878	0.189	0.105
		time	1, 16	3.739	0.071	0.189
		time × session	1, 16	0.085	0.774	0.005
	FL-DPSI	session	1, 16	2.563	0.129	0.138
		time	1, 16	3.673	0.073	0.187
		time × session	1, 16	2.617	0.125	0.141
	FL-TTS	session	1, 16	0.245	0.627	0.015
		time	1, 16	6.145	0.025	0.277
		time × session	1, 16	0.280	0.604	0.017
Anterior lateral landing task	LL-APSI	session	1, 16	1.616	0.222	0.092
		time	1, 16	6.089	0.025	0.276
		time × session	1, 16	0.002	0.963	0.0001
	LL-MLSI	session	1, 16	0.454	0.510	0.028
		time	1, 16	0.958	0.342	0.057
		time × session	1, 16	1.506	0.237	0.086
	LL-VSI	session	1, 16	0.706	0.413	0.042
		time	1, 16	2.245	0.154	0.123
		time × session	1, 16	0.319	0.580	0.020
	LL-DPSI	session	1, 16	1.140	0.302	0.067
		time	1, 16	3.347	0.086	0.173
		time × session	1, 16	0.042	0.841	0.003
	LL-TTS	session	1, 16	1.419	0.251	0.081
		time	1, 16	7.976	0.012	0.333
		time × session	1, 16	9.698	0.007	0.377
Anterior medial landing task	ML-APSI	session	1, 16	0.083	0.777	0.005
		time	1, 16	0.731	0.405	0.044
		time × session	1, 16	1.980	0.178	0.110
	ML-MLSI	session	1, 16	2.954	0.105	0.156
		time	1, 16	0.008	0.932	0.0001
		time × session	1, 16	1.213	0.287	0.070
	ML-VSI	session	1, 16	2.284	0.150	0.125
		time	1, 16	4.290	0.055	0.211
		time × session	1, 16	1.868	0.191	0.105
	ML-DPSI	session	1, 16	2.631	0.124	0.141
		time	1, 16	0.002	0.968	0.0001
		time × session	1, 16	2.897	0.108	0.153
	ML-TTS	session	1, 16	0.302	0.590	0.019
		time	1, 16	8.086	0.012	0.336
		time × session	1, 16	6.524	0.021	0.290
Y-balance test	dom-YBT_CS_	session	1, 16	0.190	0.669	0.012
		time	1, 16	10.159	0.006	0.388
		time × session	1, 16	1.337	0.264	0.077
	non-YBT_CS_	session	1, 16	18.198	0.001	0.532
		time	1, 16	148.075	0.0001	0.902
		time × session	1, 16	71.372	0.0001	0.817

Abbreviations: COP, center of pressure; COP_SA_, COP sway area (mm^2^); COP_AP_, COP maximum displacements in antero-posterior directions (mm); COP_ML_, COP maximum displacements in medio-lateral directions (mm); APSI, anterior–posterior stability index; MLSI, medial–lateral stability index; VSI, vertical stability index; DPSI, dynamic postural stability index; TTS, time to stabilization (s); non-YBT_CS_, non-dominant Y-balance composite score; dom-YBT_CS_, dominant Y-balance composite score; FL, forward landing; LL, 45° anterior lateral landing; ML, 45° anterior medial landing; non-, non-dominant; dom-, dominant.

**Table 2:** Static and dynamic postural stability scores before and after anodal or sham tDCS intervention (Mean ± SD).

Test	Parameter	Anodal Stimulation		Sham Stimulation		
		Pre	Post	Pre	Post	*p*
Static postural stability	non-COP_SA_	1387.084 ± 351.054	1300.327 ± 333.207*	1462.003 ± 422.593	1506.350 ± 383.414	0.015
	dom-COP_SA_	1453.194 ± 365.467	1283.514 ± 314.143	1275.224 ± 343.593	1396.662 ± 401.482	0.231
	non-COP_AP_	53.287 ± 7.837	52.389 ± 7.866	51.384 ± 8.598	50.339 ± 10.442	0.524
	dom-COP_AP_	53.016 ± 5.089	50.437 ± 5.913 *	48.881 ± 5.530	48.660 ± 5.254	0.322
	non-COP_ML_	52.373 ± 12.182	52.120 ± 10.282	51.593 ± 7.547	52.219 ± 7.682	0.967
	dom-COP_ML_	52.349 ± 11.661	49.352 ± 10.868	49.930 ± 7.730	50.206 ± 8.750	0.746
Forward landing task	FL-APSI	0.031 ± 0.005	0.027 ± 0.005 *	0.029 ± 0.004	0.028 ± 0.005	0.480
	FL-MLSI	0.102 ± 0.009	0.102 ± 0.007	0.100 ± 0.006	0.100 ± 0.07	0.252
	FL-VSI	0.304 ± 0.031	0.297 ± 0.028	0.296 ± 0.032	0.291 ± 0.028	0.297
	FL-DPSI	0.323 ± 0.030	0.314 ± 0.029	0.311 ± 0.028	0.309 ± 0.028	0.354
	FL-TTS	1.313 ± 0.929	1.052 ± 0.671	1.329 ± 0.692	1.199 ± 0.720	0.174
Anterior lateral landing task	LL-APSI	0.078 ± 0.009	0.076 ± 0.010	0.076 ± 0.010	0.073 ± 0.009	0.226
	LL-MLSI	0.097 ± 0.008	0.096 ± 0.009	0.095 ± 0.009	0.095 ± 0.007	0.839
	LL-VSI	0.293 ± 0.033	0.290 ± 0.027	0.288 ± 0.040	0.281 ± 0.029	0.251
	LL-DPSI	0.321 ± 0.033	0.315 ± 0.029	0.311 ± 0.037	0.306 ± 0.028	0.281
	LL-TTS	1.558 ± 0.604	1.268 ± 0.405*	1.553 ± 0.555	1.595 ± 0.699	0.044
Anterior medial landing task	ML-APSI	0.087 ± 0.011	0.085 ± 0.010	0.086 ± 0.009	0.086 ± 0.010	0.433
	ML-MLSI	0.094 ± 0.008	0.095 ± 0.010	0.092 ± 0.009	0.090 ± 0.006	0.032
	ML-VSI	0.310 ± 0.038	0.300 ± 0.031	0.293 ± 0.029	0.292 ± 0.029	0.298
	ML-DPSI	0.335 ± 0.037	0.325 ± 0.031	0.314 ± 0.028	0.319 ± 0.029	0.336
	ML-TTS	1.640 ± 0.643	1.154 ± 0.434 *	1.491 ± 0.604	1.448 ± 0.795	0.055
Y-balance test	non-YBT_CS_	101.693 ± 11.665	118.786 ± 13.464 *	102.400 ± 8.257	104.481 ± 9.266 *	0.0001
	dom-YBT_CS_	100.954 ± 11.195	104.342 ± 10.311 *	102.679 ± 7.853	103.832 ± 9.083	0.630

Abbreviations: COP, center of pressure; COP_SA_, COP sway area (mm^2^); COP_AP_, COP maximum displacements in antero-posterior directions (mm); COP_ML_, COP maximum displacements in medio-lateral directions (mm); APSI, anterior–posterior stability index; MLSI, medial–lateral stability index; VSI, vertical stability index; DPSI, dynamic postural stability index; TTS, time to stabilization (s); non-YBT_CS_, non-dominant Y-balance composite score; dom-YBT_CS_, dominant Y-balance composite score; FL, forward landing; LL, 45° anterior lateral landing; ML, 45° anterior medial landing; non-, non-dominant; dom-, dominant. * Significant changes from pre-test to post-test (*p* < 0.05).

## Data Availability

The data presented in this study are available on request from the corresponding author.
